# Traumatic Brain Injury Stimulates Neural Stem Cell Proliferation via Mammalian Target of Rapamycin Signaling Pathway Activation

**DOI:** 10.1523/ENEURO.0162-16.2016

**Published:** 2016-11-01

**Authors:** Xiaoting Wang, Pich Seekaew, Xiang Gao, Jinhui Chen

**Affiliations:** 1 Spinal Cord and Brain Injury Research Group, Indiana University, Indianapolis, IN 46202; 2 Stark Neuroscience Research Institute, Indiana University, Indianapolis, IN 46202; 3 Department of Neurological Surgery, Indiana University, Indianapolis, IN 46202

**Keywords:** mammalian target of rapamycin, neural stem/progenitor cells, proliferation, quiescence, traumatic brain injury

## Abstract

Neural stem cells in the adult brain possess the ability to remain quiescent until needed in tissue homeostasis or repair. It was previously shown that traumatic brain injury (TBI) stimulated neural stem cell (NSC) proliferation in the adult hippocampus, indicating an innate repair mechanism, but it is unknown how TBI promotes NSC proliferation. In the present study, we observed dramatic activation of mammalian target of rapamycin complex 1 (mTORC1) in the hippocampus of mice with TBI from controlled cortical impact (CCI). The peak of mTORC1 activation in the hippocampal subgranular zone, where NSCs reside, is 24–48 h after trauma, correlating with the peak of TBI-enhanced NSC proliferation. By use of a Nestin-GFP transgenic mouse, in which GFP is ectopically expressed in the NSCs, we found that TBI activated mTORC1 in NSCs. With 5-bromo-2′-deoxyuridine labeling, we observed that TBI increased mTORC1 activation in proliferating NSCs. Furthermore, administration of rapamycin abolished TBI-promoted NSC proliferation. Taken together, these data indicate that mTORC1 activation is required for NSC proliferation postinjury, and thus might serve as a therapeutic target for interventions to augment neurogenesis for brain repair after TBI.

## Significance Statement

Traumatic brain injury (TBI)-induced cell death compromises learning and memory functions in survivors by disconnecting neurocircuitries in the hippocampus, prompting an urgent need for repair strategies. Innate repair machinery driven by endogenous neural stem cells (NSCs) responds to injury but does not effect full recovery. The mechanisms of injury-induced NSC activation are elusive, further impeding possible interventions for intrinsic restoration. This study demonstrates that mTORC1 signal is activated in NSCs after trauma, and further inhibition on the mTORC1 pathway diminished the effects of injury on NSC proliferation. The results suggest that mTORC1 activation mediates TBI-enhanced NSC proliferation, providing a clinically relevant potential therapeutic target for modulation of postinjury NSC activity.

## Introduction

Traumatic brain injury (TBI), as a public health issue in the United States, causes 2.5 million hospital visits each year (Faul et al., 2010), and 52,000 patients die of severe injuries. In survivors, both physical and neurobehavioral disabilities frequently occur (Salmond and Sahakian, 2005), as well as an increased susceptibility to neurodegenerative diseases such as Alzheimer’s disease and Parkinson’s disease (Sivanandam and Thakur, 2012; Marras et al., 2014). The hippocampus is one of the brain regions most vulnerable to cell death (Colicos et al., 1996, Hicks et al., 1996; Sato et al., 2001; Hall et al., 2005), primarily acute necrotic immature neuron death in the hippocampal dentate gyrus (HDG) after trauma (Gao et al., 2008; Zhou et al., 2012). This damage results in learning and memory dysfunctions as the most common sequelae of TBI (Salmond and Sahakian, 2005; Perry et al., 2015). However, effective drugs approved by the Food and Drug Administration against cell death after TBI are unavailable. There is an urgent need for an alternative approach to neuronal replacement in the damaged hippocampus to promote cognitive functional recovery.

Adult neural stem/progenitor cells (NSCs) have been widely discovered in birds, rodents, primates, and human beings (Reynolds and Weiss, 1992; Eriksson et al., 1998; Kornack and Rakic, 1999; Nottebohm, 2002). The subgranular zone (SGZ) in the hippocampus is one of the most important niches for adult NSCs to support neurogenesis through a lifetime (Cameron and McKay, 2001; van Praag et al., 2002), contributing to learning and memory capacity (Shors et al., 2001; Kitamura et al., 2009; Akers et al., 2014; Christian et al., 2014). In both rodent models and human beings, NSC proliferation is promoted after TBI in the adult hippocampus (Dash et al., 2001; Kernie et al., 2001; Braun et al., 2002; Chirumamilla et al., 2002; Rice et al., 2003; Ramaswamy et al., 2005; Sun et al., 2005; Gao et al., 2009; Zheng et al., 2013), indicating an innate repair response of NSCs to initial injury, shown as immature neuron loss compensated in the dentate gyrus, as well as increased postinjury mature neurons in some cases (Braun et al., 2002; Sun et al., 2005, 2007; Gao and Chen, 2013; Wang et al., 2016). Moreover, neurogenesis levels after trauma correlate with injury severity (Wang et al., 2016). Functionally, neurons born after injury integrate into preexisting neurocircuitries (Emery et al., 2005; Sun et al., 2007; Villasana et al., 2015) and are responsible for spontaneous recovery (Schmidt et al., 1999; Blaiss et al., 2011; Sun et al., 2015). However, this innate repair cannot always completely compensate for cell loss, resulting in retention of permanent functional deficits in numerous TBI survivors (Prigatano, 1987; Cicerone et al., 2005). After treatment with some neurogenic agents, enhanced endogenous neurogenesis and functional improvements have been positively correlated in injured mice (Lu et al., 2003; Kleindienst et al., 2005; Lu et al., 2005; Wu et al., 2008). Taken together, these data suggest it is feasible to fully repair TBI-induced neuronal loss and restore cognitive functions by enhancing endogenous NSC-mediated neurogenesis.

NSC activity is regulated by various extracellular signals (Lie et al., 2005; Sierra et al., 2015), making it difficult to tease out the mediators of TBI-enhanced NSC proliferation. In the present study, we instead investigated the intracellular pathway to shed light on the mechanisms activating the proliferation program in NSCs after trauma. The mammalian target of rapamycin (mTOR) pathway, especially mTOR complex 1 (mTORC1), is important for coordinating extracellular signals and regulating cell proliferation (Laplante and Sabatini, 2012). It is required for maintaining the neural progenitor pool in adult and aging rodents (Paliouras et al., 2012). Additionally, activation of mTORC1 in aging mice rescued the decline of NSC proliferation and neurogenesis in the hippocampus (Romine et al., 2015). Sustained activation of mTORC1 in embryonic and neonatal NSCs leads to an imbalance of proliferation and differentiation (Magri et al., 2011; Hartman et al., 2013). Collectively, mTORC1 plays crucial roles in NSC activity modulation. After TBI, mTORC1 was activated in neurons, microglia, and astrocytes at several time points (Chen et al., 2007; Park et al., 2012). In our study, we examined mTORC1 activation in NSCs within an extended time scale up to 1 week after injury in a controlled cortical impact (CCI) model in mice. Our results indicate that mTORC1 activation in the hippocampus lasts up to 72 h after injury, whereas mTORC1 activation in NSCs mainly occurs 24–48 h after trauma. Furthermore, mTORC1 inhibition eliminated TBI-enhanced NSC proliferation in the hippocampus, demonstrating that mTORC1 signaling is required for NSC proliferation after trauma.

## Method and Materials

### Animal care

Male C57 BL/6 mice (The Jackson Laboratory, Bar Harbor, ME) were housed in a 12/12-h light/dark cycle environment. Access to food and water was provided *ad libitum*. Nestin-enhanced green fluorescent protein (EGFP) mice were kept in the same environment and were a gift from Dr. G. Enikolopov at Cold Spring Harbor Laboratories (Cold Spring Harbor, NY) as previously described (Mignone et al., 2004). All operations were performed according to protocols approved by the Indiana University Animal Care and Use Committee.

### CCI traumatic brain injury

Nine-week-old male mice (*n* = 62) were subjected to CCI injury or sham surgery. Briefly, a solution of 2.5% tribromoethanol (Avertin, Sigma-Aldrich, St. Louis, MO) was used to anesthetize the mice. The mice were fixed in a stereotaxic frame (Kopf Instruments, Tujunga, CA), and craniotomy proceeded under sterile conditions. The skin was cut and retracted, and a 4-mm craniotomy was conducted midway between the bregma and lambda sutures and laterally halfway between the central suture and the temporalis muscle. The skullcap was removed carefully with intact dura left below. Before injury, the tip of the electromagnetic impactor was adjusted and kept perpendicular to the exposed cortical surface. In the experiments, injury was set at velocity of 3.0 m/s and deformation at 1.0 mm by controlling the electromagnetic impactor. The injury site was permitted to dry before the wound was sutured. A heating pad was used during the whole surgery and recovery period to maintain the animals’ core body temperature at 36–37°C.

### Drug administration

To assess whether mTORC1 inhibition impairs TBI-enhanced NSC proliferation, mice were subjected to sham surgery or CCI injury as described above. Rapamycin (10 mg/kg; LC Laboratories, Woburn, MA) was dissolved in a solution of 5% PEG400/4% ethanol and 5% Tween 80 and administered i.p. 12, 24, 36, and 44 h after TBI (Fig. 4*A*). 5-Bromo-2′-deoxyuridine (BrdU; 100 mg/kg in saline; Sigma-Aldrich) was injected i.p. immediately after the final dose of rapamycin (Fig. 4*A*).

### Tissue processing

To assess the mTORC1 activation time course, mice were sacrificed 4, 24, 48, and 72 h and 1 week after CCI or after sham surgery. To quantify mTORC1 activation in NSCs and proliferating NSCs after TBI, mice were perfused at 24 or 48 h after trauma. To evaluate mTORC1 inhibition effects on TBI-enhanced NSC proliferation, mice were sacrificed 48 h after initial injury and the treatment described above (Fig. 4*A*). Briefly, the mice were anesthetized deeply and perfused with cold saline transcardially, followed by fixation with 4% paraformaldehyde (PFA) in PBS. Brain tissues were then collected and postfixed overnight with PFA in 4°C, followed by cryoprotection in 30% sucrose for 48 h. Serial coronal sections were cut at 30-μm thickness using a cryostat (LeicaCM 1950; Leica, Buffalo Grove, IL) and preserved at –20°C. The sections were processed for immunohistochemical analysis.

### Immunohistochemistry

First, free-floating sections were washed in PBS three times, followed by incubation in blocking buffer (0.1% Triton X-100, 1% bovine serum albumin, 5% normal goat serum in PBS) for 1 h. Then sections were incubated with primary antibody overnight at 4°C, washed for three times in PBS, and then incubated with secondary antibody for 2 h at 4°C. After 4′,6-diamidino-2-phenylindole (DAPI) treatment for 2 min, sections were washed again with PBS three times and mounted with Fluoromount-G (SouthernBiotech, Birmingham, AL). For BrdU incorporation, sections were treated before standard blocking protocol as follows: incubation with 2N HCl was performed at room temperature for 1 h, followed by soak in 0.1 m (pH 8.4) borate buffer for 10 min. After three washes in PBS, sections were processed according to standard protocol in blocking solution. The primary antibodies and their final concentrations used in the experiments were as follows: anti-BrdU (1:200, rat; AbD Serotec, Raleigh, NC), anti-pS6 (1:200, rabbit; Cell Signaling Technology, Danvers, MA), anti-GFP (1:1000, chicken; Abcam, Cambridge, MA), and anti-Sox2 (1:1000, goat; R&D Systems, Minneapolis, MN). Secondary antibodies were from Jackson ImmunoResearch Laboratories (West Grove, PA) and applied in a 1:1000 dilution.

### Immunoblotting

To assess mTORC1 activation in the whole hippocampus, mice were sacrificed at 4, 24, 48, and 72 h and 1 week after CCI or after sham surgery. The ipsilateral hippocampi were collected, homogenized with ice-cold Triton lysis buffer (1% Triton X-100, 20 mm Tris-HCL, 150 mm NaCl, 5 mm EGTA, 10 mm EDTA, and protease inhibitor cocktail [Roche, Basel, Switzerland]), and centrifuged for 30 min at 14,000 rpm, 4°C. Protein concentration was determined by a modified Lowry assay (Bio-Rad, Hercules, CA). The same amount of protein in each sample was loaded and run on SDS/PAGE. After electrotransfer to nitrocellulose membranes at 30 V overnight at 4°C, the membrane was incubated in PBS with 5% nonfat milk at room temperature for 1 h and probed with antibodies against S6 (1:1000, rabbit, Cell Signaling Technology), β-actin (1:000, mouse, Abcam), and pS6 (1:200, rabbit, Cell Signaling Technology) overnight at 4°C. Secondary antibodies were used at a dilution of 1:5000. The membrane was washed three times and rinsed in TBST. Finally, proteins were detected with ECL substrates (Bio-Rad), and images were acquired using normal image scanning methods for colorimetric detection.

### Cell counting

Brain sections were simultaneously processed for immunohistochemistry. An inverted fluorescent microscope system (Axiovert 200M, Zeiss, Jena, Germany) was used to analyze the sections. Series of every sixth section (30 μm thickness, 180 μm apart, 12–16 sections in each animal) from covered whole hippocampus were analyzed in a double-blind fashion. Double-positive cells were counted under the microscope with a 40× objective through the whole series. We used one marker as an indicator; when a positive cell showed in the field, we switched to the channel matching second marker signal. If the target cell also had been labeled, we counted it as a double-positive cell. Triple-positive cells were similarly counted under the microscope through the whole series. We used one marker as an indicator; when a positive cell showed in the field, we switched to the channel matching second marker signal. If the target cell also had been labeled, we again switched to the channel matching third marker signal. Then if the target cell again had been labeled, we counted it as a triple-positive cell.

In all experiments, double- and triple-positive cells were quantified in a double-blind fashion using a profile count method. Briefly, every single colabeled cell (even partial cells at the borders of sections) was counted in multiplanes throughout the region of interest in the 30-μm section. Contours of granule cell layer (GCL) were created in Zeiss software (AxioVision v4.8) to measure the volume of GCL. The cell density was calculated by dividing total cell number by the GCL volume, thus expressed as average number/mm^3^.

### Microscopy

The inverted fluorescent microscope (Zeiss Axiovert 200M) used for cell counting and image capture was combined with an ApoTome structured-illumination attachment (Zeiss). A digital camera (Zeiss Axio Cam MRc5) interfaced with the microscope was controlled by a computer. Representative images were captured and stacked as a cut view image in software (AxioVision v4.8). The images were assembled and labeled in Photoshop 7.0 (Adobe Systems, San Jose, CA).

### Statistical analysis

Quantification of target cells is shown as average ± SD. Data were analyzed via appropriate type of ANOVA followed by Fisher’s least significant difference test. Superscript letters listed with *p*-values correspond to the statistical tests shown in Table 1. Statistical analysis was done using SPSS software (IBM, Armonk, NY). Significance was set at *p* < 0.05.

**Table 1.** Statistical analysis.

**Line**	**Data structure**	**Type of test**	**Power**
a	Normal distribution	One-way ANOVA	0.896
b	Normal distribution	One-way ANOVA	0.941
c	Normal distribution	One-way ANOVA	1.000
d	Normal distribution	One-way ANOVA	1.000
e	Normal distribution	One-way ANOVA	0.999
f	Normal distribution	One-way ANOVA	0.889
g	Normal distribution	One-way ANOVA	0.986
h	Normal distribution	One-way ANOVA	0.997
i	Normal distribution	Two-way ANOVA	0.988 for injury 0.732 for treatment 0.478 for interaction

## Results

### mTORC1 signaling activation in the whole hippocampus after TBI

NSCs have long been reported to respond to brain injuries, including stroke, seizure, and TBI (Parent et al., 1997, 2002; Dash et al., 2001; Kernie et al., 2001; Yagita et al., 2001; Braun et al., 2002; Chirumamilla et al., 2002; Rice et al., 2003; Ramaswamy et al., 2005; Sun et al., 2005; Yamashita et al., 2006; Gao et al., 2009; Zheng et al., 2013). After TBI, enhanced NSC proliferation in the hippocampus has been observed regardless of injury model and animal model (Dash et al., 2001; Kernie et al., 2001; Braun et al., 2002; Chirumamilla et al., 2002; Rice et al., 2003; Ramaswamy et al., 2005; Sun et al., 2005; Gao et al., 2009; Zheng et al., 2013). It was further demonstrated that quiescent NSCs are the subgroup mainly activated by TBI (Gao et al., 2009). However, the molecular mechanism underlying the phenomenon remains elusive, impeding development of interventions aimed at promoting neurogenesis by further enhancing NSC proliferation after trauma. The mTOR signaling pathway, especially mTORC1, is known to be involved in NSC activity regulation in embryonic (Magri et al., 2011), neonatal (Hartman et al., 2013), adult (Paliouras et al., 2012), and aging (Paliouras et al., 2012; Romine et al., 2015) rodents, and its activation has also been reported after TBI in the hippocampus (Chen et al., 2007; Park et al., 2012), so we proposed that mTORC1 signaling mediates TBI-enhanced NSC proliferation.

**Figure 1.**
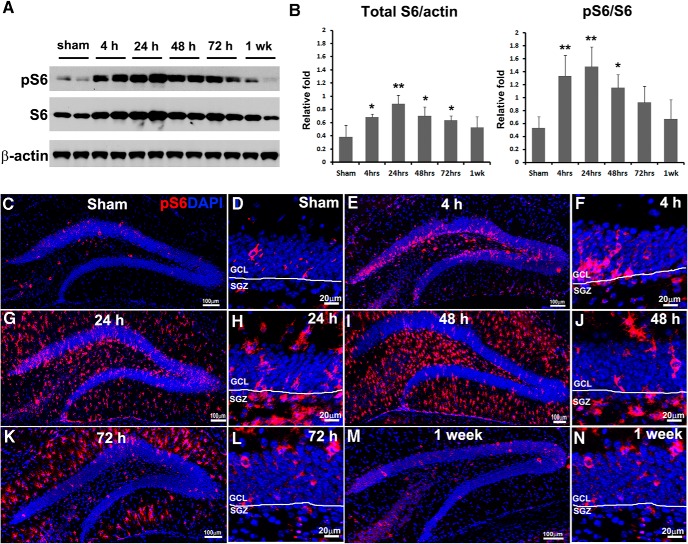
TBI activates mTORC1 signaling in the hippocampus. Mice received a moderate CCI at the age of 9 weeks and were sacrificed at 4, 24, 48, and 72 h and 1 week after injury as well as after sham injury (*n* = 3 for each group). ***A***, Immunoblotting with antibodies against pS6, S6, and β-actin shows mTORC1 signaling activation in the hippocampus. ***B***, Quantification of blots shown in ***A*** (*p < 0.05, **p < 0.01). ***C–N***, Immunostaining with antibody against pS6 (red) shows mTORC1 signaling activation in the HDG after sham surgery (***C***) and 4 h (***E***), 24 h (***G***), 48 h (***I***), 72 h (***K***), and 1 week (***M***) after CCI, and in the SGZ at corresponding time points (***D***, ***F***, ***H***, ***J***, ***L***, ***N***), respectively. DAPI staining shows the structure of HDG.

To demonstrate this hypothesis, we induced moderate TBI in adult mice by a CCI injury model and evaluated mTORC1 signal activation in the whole hippocampus at different time points postinjury. Adult mice were sacrificed 4, 24, 48, and 72 h and 1 week after CCI or after sham surgery, and ipsilateral hippocampi were subjected to immunoblotting. We evaluated the total amount and phosphorylated form of ribosomal protein S6 (a widely used marker for mTORC1 activation; Laplante and Sabatini, 2012; Fig. 1*A*). After trauma, total S6 was elevated rapidly 4 h postinjury (*F*_(5,12)_ = 5.108, *p* = 0.017^a^; Fig. 1*B*), reached a peak at 24 h (*F*_(5,12)_ = 5.108, *p* = 0.001^a^; Fig. 1*B*), stayed at high levels at 48 h (*F*_(5,12)_ = 5.108, *p* = 0.012^a^; Fig. 1*B*) and 72 h (*F*_(5,12)_ = 5.108, *p* = 0.043^a^; Fig. 1*B*), and returned back to normal level by 1 week after injury (*F*_(5,12)_ = 5.108, *p* = 0.230^a^; Fig. 1*B*). Meanwhile, the phosphorylation level (pS6) was dramatically increased at 4 h (*F*_(5,12)_ = 6.000, *p* = 0.003^b^; Fig. 1*B*), further elevated at 24 h (*F*_(5,12)_ = 6.000, *p* = 0.001^b^; Fig. 1*B*), stayed at high levels at 48 h (*F*_(5,12)_ = 6.000, *p* = 0.013^b^; Fig. 1*B*), remained slightly higher than sham level at 72 h (*F*_(5,12)_ = 6.000, *p* = 0.091^b^; Fig. 1*B*), and returned comparable to baseline by 1 week (*F*_(5,12)_ = 6.000, *p* = 0.537^b^; Fig. 1*B*). Together, the data suggest that the mTORC1 signal was activated in the hippocampus mainly at 4, 24, and 48 h after TBI.

### mTORC1 signaling activation in the HDG after TBI

To assess temporal-spatial mTORC1 activation in the HDG, adult mice were sacrificed 4, 24, 48, and 72 h and 1 week after CCI or sham surgery. The epicenter section from each animal was processed for immunostaining with antibody against pS6. In sham animals, a limited number of pS6-positive cells were mainly observed in the GCL and few in the hilus, indicating a baseline of mTORC1 activity in the HDG (Fig. 1*C*). After TBI, we observed a wave of mTORC1 activation in the HDG starting no later than 4 h, maintaining a high level of activation at least until 72 h, and returning to sham level by 1 week postinjury (Fig. 1*E*, *G*, *I*, *K*, *M*).

Meanwhile, mTORC1 activation showed different patterns regarding signal location in subregions in the HDG at different time points. At 4 h after TBI, increased pS6-positive cells were predominantly restricted to the GCL (Fig. 1*E*), indicating a rapid response in granule neurons to the initial insult. At 24 h after trauma, the pS6-positive cells not only further accumulated in the GCL, but also vastly spread to the molecular layer (ML) and hilus (Fig. 1*G*). At 48 h after CCI, the pS6 signal reached a peak, and positive cells were mainly observed in the ML and hilus but few in the GCL (Fig. 1*I*), implying strong mTORC1 activation in reactive glia, especially reactive microglia, at this time point according to our unpublished data. At 72 h after trauma, mTORC1 activation started decreasing, and the remaining positive cells were mostly in the ML (Fig. 1*K*), largely attributable to reactive astrocytes based on our unpublished data. By 1 week after TBI, the pS6 signal was back to sham level and again showed sporadic activation mainly in the GCL (Fig. 1*M*). Collectively, we detected a time-dependent and location-shifted mTORC1 activation pattern in the HDG after TBI, indicating mTORC1 involvement in various responses to CCI in multiple cell types.

### Time course of mTORC1 signaling activation in the SGZ after TBI

To further assess whether mTORC1 is activated in the NSCs after TBI, we focused on pS6 signal in the SGZ, where adult NSCs locate in the hippocampus (Faigle and Song, 2013). In sham animals, we again observed pS6-positive cells mainly in the GCL (Fig. 1*D*). At 4 h after CCI, increased pS6 signal was seen primarily in the GCL but not SGZ (Fig. 1*F*). At 24 h postinjury, a dramatic increase of pS6-positive cells showed up in the SGZ (Fig. 1*H*), as well as in the ML and hilus. At 48 h after CCI, pS6-positive cells in the SGZ decreased compared with 24 h, but were still more numerous than in sham surgery (Fig. 1*J*). At 72 h and 1 week after TBI, mTORC1 activation in the SGZ was limited and comparable to that of sham animals (Fig. 1*L*, *N*). Taken together, our data enabled us to narrow down activation of mTORC1 in the SGZ predominantly to 24 and 48 h after initial insult, suggesting the potential time course of mTORC1 activation in NSCs. The duration of mTORC1 activation correlates with TBI-enhanced NSC proliferation (Gao et al., 2009), suggesting possible involvement of mTORC1 activity in TBI-enhanced NSC proliferation.

### TBI activates mTORC1 signaling in NSCs

**Figure 2.**
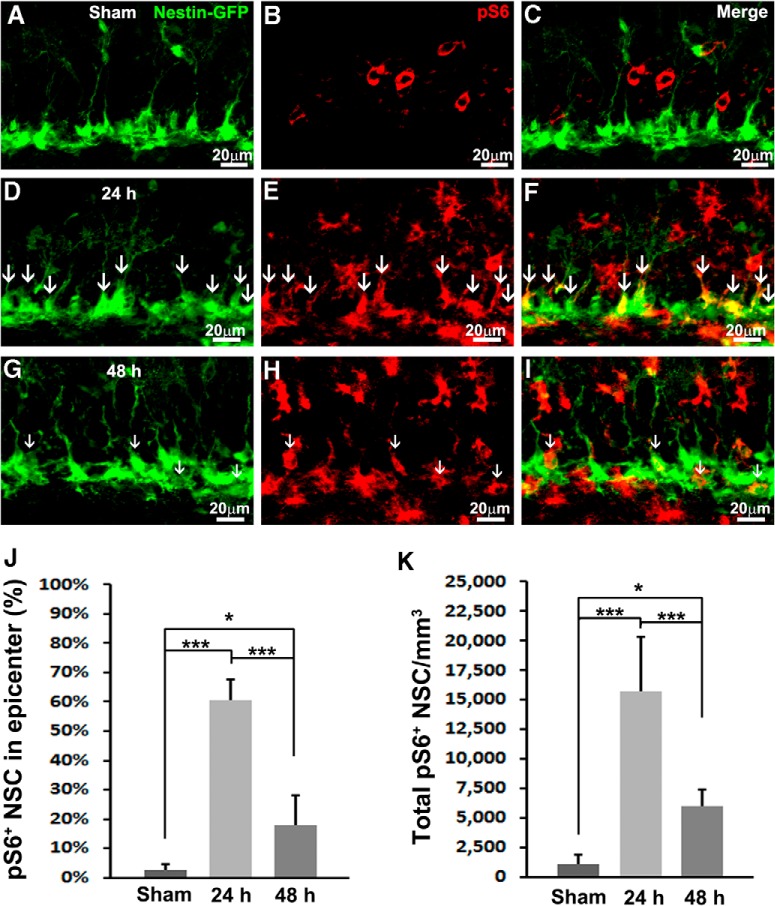
TBI activates mTORC1 signaling in NSCs. Mice received a moderate CCI at the age of 9 weeks and were sacrificed at 24 or 48 h after injury as well as after sham injury (*n* = 5 for each group). ***A–I***, Immunostaining with antibodies against GFP (green) and pS6 (red) shows mTORC1 signaling activation in NSCs (white arrows) in sham animals (***A–C***) 24 h (***D–F***) and 48 h (***G–I***) after CCI in the subgranular zone. ***J***, Quantification of total pS6-positive NSCs after sham surgery and 24 and 48 h after CCI, respectively. ***K***, Quantification of percentage of pS6-positive NSCs in the epicenter after sham surgery and 24 and 48 h after CCI, respectively (*p < 0.05, **p < 0.01, ***p < 0.001).

To accurately determine whether mTORC1 is activated in NSCs, we colabeled pS6 with an NSC marker. Additionally, we took advantage of a Nestin-GFP transgenic mouse, in which NSCs ectopically express green fluorescent protein (GFP; Mignone et al., 2004; Gao et al., 2009). We hardly observed NSCs costaining with pS6 in the SGZ of sham-treated animals (Fig. 2*A*–*C*). Quantification showed that 2.6 ± 2.1% of NSCs were pS6 positive at the epicenter (Fig. 2*J*). When we further examined NSCs across the whole hippocampus, only 1096 ± 805/mm^3^ of total NSCs in the whole hippocampus were colabeled with pS6 (Fig. 2*K*). These results confirmed our prior notion that mTORC1 activity in the NSCs is very low in the sham-treated animals.

At 24 h after TBI, the number of pS6-positive cells in the SGZ was dramatically increased (Fig. 2*E*) and largely labeled NSCs (Fig. 2*D–F*, white arrows). Quantification showed that mTORC1 is active in 60.7 ± 6.8% of total NSCs in the epicenter (*F*_(2,12)_ = 35.077, *p* < 0.001^c^; Fig. 2*J*). In all, mTORC1 signaling is active in 15750 ± 4620/mm^3^ NSCs across the whole hippocampus (*F*_(2,12)_ = 85.257, *p* < 0.001^d^; Fig. 2*K*), indicating a dramatic 15-fold increase compared with sham control.

At 48 h after CCI, the number of pS6-positive cells was still very high (Fig. 2*H*), whereas labeling in NSCs decreased (Fig. 2*G–I*, white arrows). At the epicenter, the percentage of mTORC1-positive NSCs also dropped to 17.7 ± 10.4% (*F*_(2,12)_ = 35.077, *p* < 0.001^c^ vs. 24 h, *p* = 0.017^c^ vs. sham; Fig. 2*J*). The number of NSCs with active mTORC1 across the whole hippocampus rapidly decreased to 5987 ± 1348/mm^3^ (*F*_(2,12)_ = 85.257, *p* < 0.001^d^ vs. 24 h) but was still much higher than basal level (*F*_(2,12)_ = 85.257, *p* = 0.044^d^ vs. sham; Fig. 2*K*). In all, we observed a rapid and robust activation of mTORC1 in NSCs 24 h after TBI that lasted at least to 48 h after trauma.

### TBI activates mTORC1 signaling in proliferating NSCs

**Figure 3.**
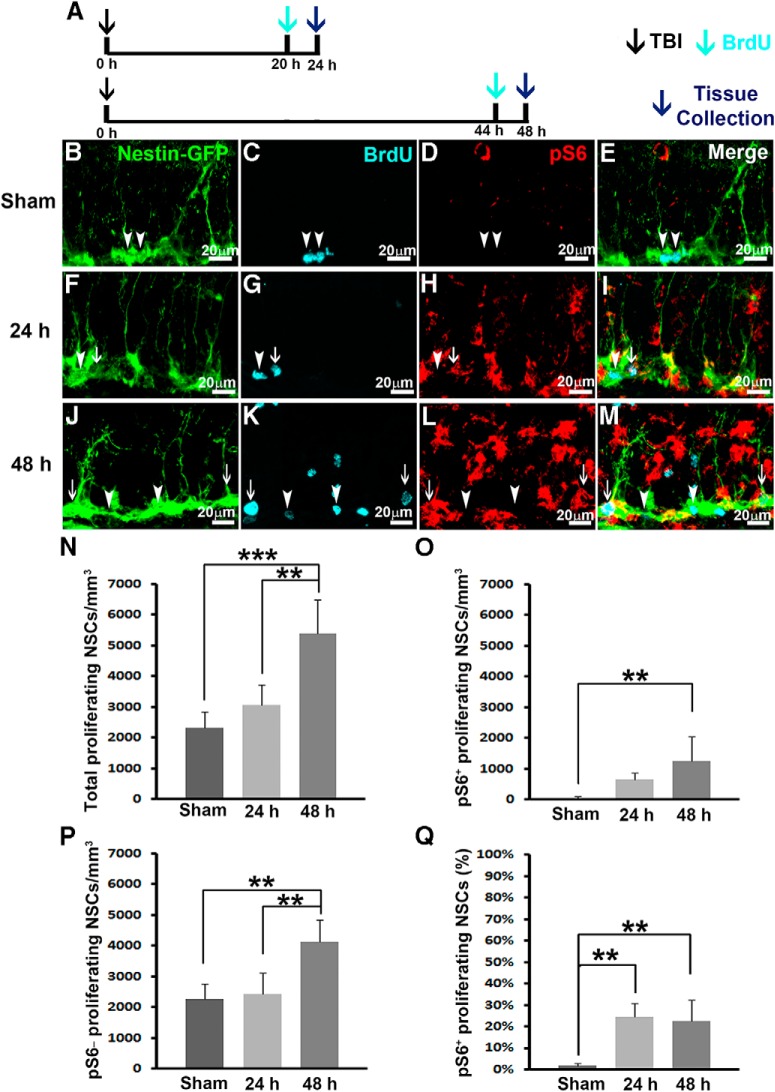
TBI activates mTORC1 signaling in proliferating NSCs. Mice received a moderate CCI at the age of 9 weeks and were sacrificed at 24 or 48 h after injury as well as after sham injury (*n* = 5 for each group). A dose of BrdU was administered 4 h before perfusion. ***A***, Experimental strategy. ***B–M***, Immunostaining with antibodies against GFP (green), BrdU (cyan), and pS6 (red) shows proliferating NSCs with (white arrows) or without (arrowheads) mTORC1 signaling activation in sham animals (***B–E***), 24 h (***F–I***) and 48 h (***J–M***) after CCI in the subgranular zone. ***N–P***, Quantification of total proliferating NSCs (***N***), total pS6-positive proliferating NSCs (***O***), and total pS6-negative proliferating NSCs (***P***) after sham surgery and 24 and 48 h after CCI, respectively. ***Q***, Quantification of percentage of pS6-positive proliferating NSCs after sham surgery and 24 and 48 h after CCI, respectively (*p < 0.05, **p < 0.01, ***p < 0.001).

Previously, it was proven that TBI transiently promoted NSC proliferation around 24–48 h postinjury (Gao and Chen, 2013). The molecular mechanisms that mediate TBI-enhanced NSC proliferation are completely unknown. The timing of mTORC1 signaling activation correlates well with NSC proliferation after TBI, strongly suggesting that mTORC1 signaling activation is involved in regulating TBI-induced NSC proliferation. Thus we further assessed whether mTORC1 signaling is activated in proliferating NSCs. BrdU injection (i.p., 100 mg/kg) was given 4 h before sacrifice (Fig. 3*A*), and the proliferating cells during this 4 h period were pulse-labeled. Series of every sixth section were processed for triple immunostaining to assess mTORC1 activity in proliferating NSCs.

In sham-treated animals, only 2320 ± 513/mm^3^ NSCs were proliferating, as pulse-labeled by BrdU (Fig. 3*B*, *C*, *E*, white arrowheads, and Fig. 3*N*). Among these proliferating NSCs, only 49 ± 40/mm^3^ of them were pS6 positive (Fig. 3*O*), representing only 1.9 ± 1.2% of total proliferating NSCs that were pS6 positive (Fig. 3*Q*). These data indicate that only a small population of NSCs are proliferating in the hippocampus of mice receiving sham surgery, and the activity of mTORC1 signaling at these proliferating NSCs is extremely low.

Twenty-four hours after receiving moderate CCI injury, 3070 ± 658/mm^3^ NSCs were proliferating in the hippocampus of the mice (Fig. 3*F*, *G*, *I*, white arrowhead and arrow, and Fig. 3*N*). However, TBI significantly activated mTORC1 signaling in the hippocampus (Fig. 3*H*). Of the proliferating NSCs, 649 ± 229/mm^3^ or 24.7 ± 6.2% were pS6 positive (Fig. 3*F–I*, white arrow, and Fig. 3*O*, *Q*), whereas the rest of the proliferating NSCs were pS6 negative (Fig. 3*F–I*, white arrowhead). These results indicate TBI did not dramatically alter NSC proliferation at 24 h compared with sham animals (*F*_(2,12)_ = 20.432, *p* = 0.328^e^; Fig. 3*N*), which has been demonstrated before (Gao and Chen, 2013); however, TBI promoted a 13-fold increase of mTORC1 activation in the proliferating NSCs 24 h after TBI (*F*_(2,12)_ = 17.037, *p* = 0.001^h^; Fig. 3*Q*).

Forty-eight hours after receiving moderate CCI injury, 5396 ± 1092/mm^3^ NSCs were proliferating in the hippocampus of the mice (Fig. 3*J*, *K*, *M*, arrowheads and arrows, and Fig. 3*N*). TBI dramatically increased the proliferation of NSCs in the hippocampus at this time point (*F*_(2,12)_ = 20.432, *p* < 0.001^e^), agreeing with a previous report that TBI transiently promoted NSC proliferation 48 h after TBI (Gao and Chen, 2013). TBI also significantly promoted mTORC1 signaling in the proliferating NSCs (1260 ± 798/mm^3^, *F*_(2,12)_ = 7.954, *p* = 0.005^f^ vs. sham; Fig. 3*J–M*, white arrows, and Fig. 3*O*). These data indicate that TBI significantly promotes NSC proliferation (Fig. 3*N*) and enhanced mTORC1 activity in the proliferating NSCs 48 h after injury (Fig. 3*O*, *Q*). In all, we observed a dramatically increased level of mTORC1 activation in proliferating NSCs 24 and 48 h after TBI, and TBI transiently promoted NSC proliferation beginning 48 h after TBI. The sequence of mTORC1 activation and NSC proliferation strongly suggest the possible involvement of mTORC1 signaling pathway activation in TBI-enhanced NSC proliferation.

### Inhibition of mTORC1 signaling eliminates TBI-enhanced NSC proliferation

**Figure 4.**
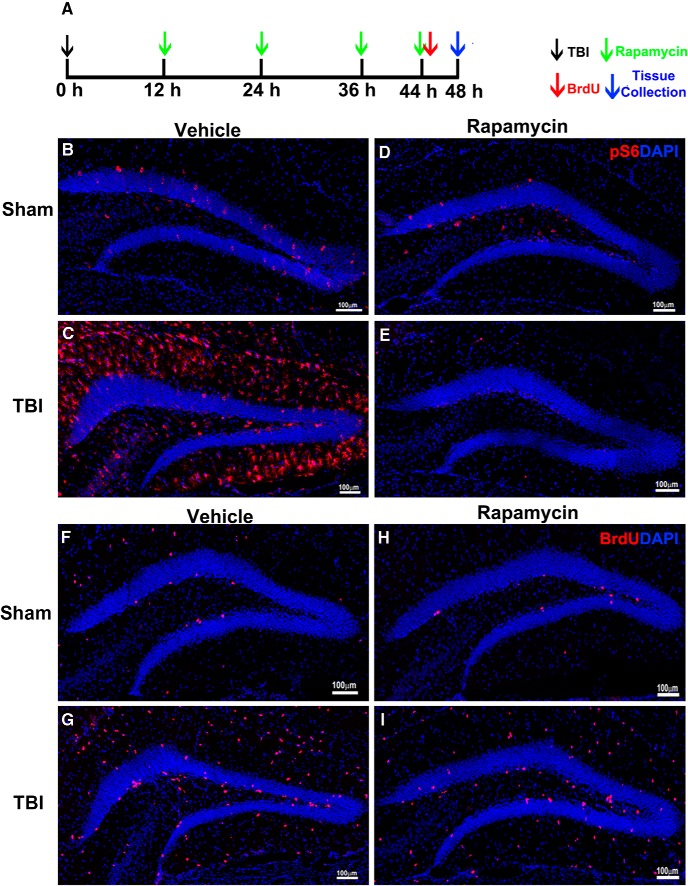
Rapamycin treatment inhibits mTORC1 signaling and cell proliferation in the hippocampus after TBI. ***A***, Experimental strategy. ***B–E***, Immunostaining with antibody against pS6 (red) shows mTORC1 signaling activity after rapamycin or vehicle treatment in sham animals or 48 h after CCI. ***F–I***, Immunostaining with antibodies against BrdU (red) shows cell proliferation in the HDG after rapamycin or vehicle treatment in sham animals or 48 h after CCI. DAPI staining shows structure of HDG.

To further confirm that mTORC1 activation is required for TBI-enhanced NSC proliferation, we treated TBI animals with rapamycin, a well-established mTORC1 inhibitor, and evaluated NSC proliferation 48 h after TBI. To fully block mTORC1 activity, four injections of rapamycin (i.p., 10 mg/kg) or vehicle were given at 12, 24, 36, and 44 h after TBI or sham surgery. Immediately after the last rapamycin injection, a dose of BrdU (i.p., 100 mg/kg) was given to label cell proliferation, and then animals were perfused at 48 h after injury (Fig. 4*A*).

To evaluate mTORC1 inhibition, the epicenter section from each animal was processed for pS6 staining. The low-level mTORC1 activation in sham animals was not apparently affected by rapamycin (Fig. 4*B*, *D*), whereas the originally strong mTORC1 activation in the HDG was dramatically abolished in TBI animals 48 h after surgery (Fig. 4*C*, *E*). pS6 staining demonstrated successful mTORC1 inhibition in the HDG after trauma. To assess overall cell proliferation, the epicenter section from each animal was processed for BrdU staining. An obvious decrease of BrdU-positive cells in the HDG was observed in both rapamycin-treated sham and TBI animals compared with vehicle groups (Fig. 4*F*–*I*). These data indicate that inhibition of mTORC1 dramatically reduces cell proliferation in the hippocampus.

**Figure 5.**
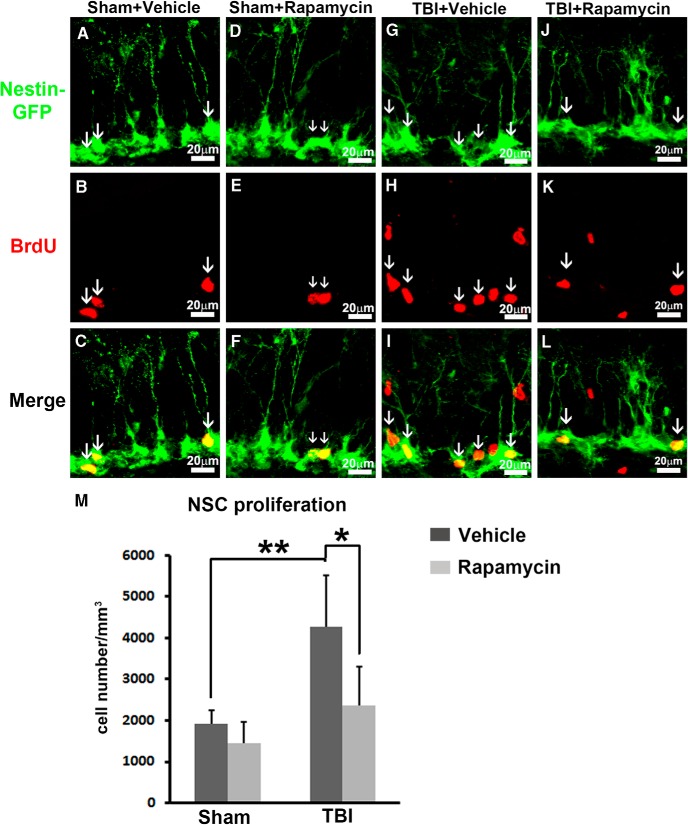
Inhibition of mTORC1 signaling ablates TBI-enhanced NSC proliferation. Mice were treated with the same procedure as in Figure 4*A* (*n* = 5 for each group). ***A–L***, Immunostaining with antibodies against GFP (green) and BrdU (red) shows NSC proliferation (white arrows) in the SGZ after rapamycin or vehicle treatment in sham animals and 48 h after CCI. ***M***, Quantification of NSC proliferation in the SGZ after rapamycin or vehicle treatment in sham animals and 48 h after CCI (*p < 0.05, **p < 0.01, ***p < 0.001).

Different types of cells, including glia and NSCs, proliferate after TBI. To further determine whether inhibition of mTORC1 affects NSC proliferation, double immunostaining with an NSC marker was performed (Fig. 5). In sham control animals treated with vehicle, there were 1925 ± 313/mm^3^ NSCs proliferating (Fig. 5*A–C*, arrows, and Fig. 5*M*), which was slightly decreased by rapamycin treatment to 1436 ± 519/mm^3^, without significant difference (*F*_(1,19)_ = 7.543, *p* = 0.956^9^; Fig. 5*D–F*, *M*). After TBI, NSC proliferation was dramatically increased to 4260 ± 1251/mm^3^ (*F*_(1,19)_ = 20.233, *p* = 0.001^i^ vs. sham + vehicle; Fig. 5*G–I*, *M*), whereas rapamycin treatment abolished the enhanced effect on NSC proliferation (2375 ± 920/mm^3^, *F*_(1,19)_ = 7.543, *p* = 0.018^i^ vs. TBI + vehicle; Fig. 5*J–L*, *M*). Altogether, our data suggest that inhibition of mTORC1 signaling eliminates TBI-enhanced NSC proliferation, indicating that mTORC1 signaling pathway activation is required for TBI-enhanced NSC proliferation.

## Discussion

TBI induces dramatic cell death in the hippocampus, which contributes to vast disconnections of local neurocircuitries and subsequent neurobehavioral dysfunctions. So far, no Food and Drug Administration–approved drug against neuronal loss caused by TBI is available, and effective neuroprotective or alternative neural repair approaches are urgently needed. NSCs in the hippocampus represent the potential of neuroregeneration in the adult brain and hold great promise for neuronal replacement after trauma (Kuhn et al., 1996; Shapiro and Ribak, 2005; Ming and Song, 2005; Zhao et al., 2006). It has been widely reported that NSC proliferation increases after TBI (Dash et al., 2001; Kernie et al., 2001; Braun et al., 2002; Chirumamilla et al., 2002; Rice et al., 2003; Ramaswamy et al., 2005; Sun et al., 2005; Gao et al., 2009; Zheng et al., 2013), as well as production of mature neurons in some circumstances (Sun et al., 2005, 2007; Wang et al., 2016). This phenomenon shows the regenerative potential of adult brain by neurogenic response of NSCs after TBI. However, the increased NSC proliferation does not always result in enhanced neurogenesis (Gao and Chen, 2013; Wang et al., 2016). Therefore, the innate response is not always strong enough to fully compensate for neuronal loss, and thus approaches are needed to further promote NSC proliferation after trauma. Unfortunately the molecular mechanisms underlying TBI-enhanced NSC proliferation are currently unknown, largely impeding its application.

**Figure 6.**
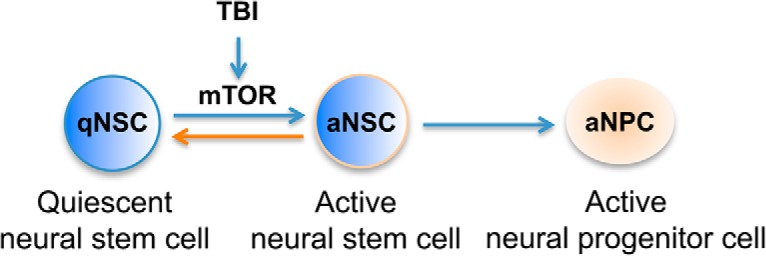
Reactivation of quiescent neural stem cells after TBI. Quiescence differs from other nondividing states in that it can be reverted into proliferation. TBI activates mTOR in NSCs, which is essential for NSCs to exit quiescence and enter proliferation.

Neural stem/progenitor cells in the hippocampus can be categorized into at least two subtypes based on morphology, protein marker expression, and pattern of division (Seri and García-Verdugo, 2001; Seaberg and van der Kooy, 2002; Filippov et al., 2003; Mignone et al., 2004; Bull and Bartlett, 2005; Encinas et al., 2006, 2008; Encinas and Enikolopov, 2008). Only a small fraction (1.73%) of radial glia-like type I NSCs can be labeled with BrdU after a short (4-h) pulse (Gao et al., 2009), indicating that they are normally quiescent (Mignone et al., 2004; Encinas et al., 2006); hence they are designated as quiescent neural stem cells (qNSCs) or active neural stem cells (aNSCs) depending on whether they are active in proliferation. Under basal conditions, qNSCs play the role of stem cells, whereas aNSCs undergo asymmetric divisions to generate small round or oval-shaped type II neural progenitor cells (NPCs). These progeny cells undergo a series of symmetric divisions and can be labeled with BrdU with high frequency. After a short (4-h) pulse, 14.99% of NPCs can be labeled with BrdU (Gao et al., 2009); they are described as amplifying neural progenitor cells (aNPCs; Mignone et al., 2004; Encinas et al., 2006). It has been recently found that TBI activates quiescent qNSCs. The percentage of proliferating qNSCs was significantly increased from 1.73 ± 0.16% to 3.76 ± 0.48%, a 2.17-fold difference (Gao et al., 2009), indicating that TBI may release a subpopulation of qNSCs from quiescent status and promote them to reenter the cell cycle, thus becoming active NSCs (aNSCs). The activation of qNSCs by TBI is transient. The NSCs reverted back to quiescence in a few days after TBI, indicating that quiescence and proliferation are reversible (Fig. 6). The molecular mechanisms that activate qNSCs after TBI are completely unknown.

Cellular quiescence is a reversible nonproliferating state and outside of an active cell cycle (also called G0; Infante et al., 2008; Coller, 2011). Quiescence serves an essential role in preserving stem cell function until the stem cell is needed in tissue homeostasis or repair. Cell quiescence and proliferation are tightly regulated to maintain tissue homeostasis (Coller, 2011; Hilpert et al., 2014; Lin et al., 2014; Rodgers et al., 2014). The reactivation of qNSCs into proliferation is crucial for tissue repair and regeneration. This reactivation not only exists in neural stem cells, but it also occurs in other tissue stem cells such as fibroblasts and hematopoietic stem cells (Rumman et al., 2015). Studies in the past few years have revealed that, instead of being a passive state, quiescence is actively maintained in the cell (Spencer et al., 2013; Hilpert et al., 2014). Quiescent cells are transcriptionally active. The extent to which stem cells can regulate quiescence and reactivation is very poorly understood. Our present study, in a rodent CCI model, demonstrates that mTORC1 activation is required for activating quiescent NSCs by TBI. Our result demonstrates that mTOR signaling is a key regulator of the stem cell fate choice. We provide a model of reactivation of quiescent neural stem cells for brain repair after injury (Fig. 6). This reactivation can potentially be targeted for interventions on NSC activity and concomitant neuronal replacement for functional recovery after trauma.

As a fundamental pathway in all types of cells, mTOR signaling is involved in many important cellular events, including metabolism, cell growth, cell cycle progression, and cell survival (Laplante and Sabatini, 2012). Specifically in the nervous system, particularly in NSCs, mTOR signaling, especially mTORC1, is also significant for balancing self-renewal and differentiation. It has been reported that constitutive hyperactivity of mTORC1 signaling is responsible for deregulated NSCs activity in the embryonic stage and leads to tuberous sclerosis development (Magri et al., 2011). In postnatal development, mTORC1 activity is also required for NSCs, especially in maintenance of the transit amplifying neural progenitor pool (Paliouras et al., 2012). Decline of NSC proliferation in aging rodents can also be restored by activating mTORC1 activity (Romine et al., 2015). Collectively, mTORC1 plays significant roles in regulating NSC activity, particularly in regulating NSC proliferation. After TBI, activation of mTORC1 signaling in the injured cortex and hippocampus has been noticed for a while (Chen et al., 2007; Park et al., 2012); however, the role of mTORC1 activation remains elusive. Initially, mTORC1 was proven to mediate apoptotic neuronal death within hours after TBI, and early inhibition of mTORC1 signaling by rapamycin pretreatment has been shown to alleviate motor deficits and cognitive impairments 3 days after trauma (Park et al., 2012; Ding et al., 2015). Later on, mTORC1 activation in microglia and astrocytes postinjury has further been noticed, thus bringing about the idea that roles of mTORC1 in TBI pathogenesis include inducing neuroinflammation and promoting astrogliosis, which was also reversed by rapamycin administration (Ding et al., 2014; Nikolaeva et al., 2015). Besides, genetic activation of mTORC1 by upstream negative regulator inactivation worsens cognitive performances after TBI in rodents (Rozas et al., 2015). Recently, Butler et al. (2015) pointed out a regulatory role of mTORC1 in neurogenesis and synaptic reorganization after TBI, which was potentially involved in post-traumatic epileptogenesis. Altogether, mTORC1 activation was considered a deleterious event after TBI; thus it was targeted in most studies. However, another study demonstrated that rapamycin treatment worsened cognitive deficits instead (Zhu et al., 2014). Our unpublished data also indicate that mTOR activation is required for survival of spared neurons in the injured cortex. The exact role of mTOR in TBI pathophysiology has yet to be determined. In our present study, we focused on the regulatory role of mTORC1 especially in NSC proliferation after moderate TBI using a CCI model in rodents. We illustrated that mTORC1 activation in NSCs in the hippocampus was delayed to 24–48 h after initial injury compared with 4 h after trauma in mature neurons reported by other studies (Chen et al., 2007; Ding et al., 2015). In addition, inhibition by rapamycin abolished TBI-enhanced NSC proliferation in the hippocampus 48 h after moderate CCI. This study demonstrates a beneficial role of mTORC1 in neuroplasticity by mediating NSC proliferation after TBI. Although some studies proved that inhibition of mTORC1 activity after TBI by pre- or post-treatment with rapamycin improved motor functions and cognitive outcomes at the acute phase after TBI, the present results suggest that these regimens may come at the expense of compromising long-term neuroregeneration. In contrast, short-term enhancing mTOR activation may increase neurogenesis and have a long-term effort in improving learning and memory functions after TBI. In all, the role of mTOR activation is specific to cell type and time course in TBI pathophysiology. Thus a detailed spatial-temporal profile of mTOR activation is required, before clearly determining the therapeutic potential of inhibition or activation of its activity, as well as when and where mTOR activity should be modulated in terms of beneficial neurobehavioral outcomes in TBI patients. Meanwhile, as a multifunctional signaling pathway, mTOR may not be a good target for therapeutic applications. Further investigations are needed to determine specific downstream targets of mTOR in different scenarios of individual cell types, namely mature neuron apoptosis, NSC proliferation, microglia activation–mediated neuroinflammation, and astrogliosis.

## Synthesis

The decision was a result of the Reviewing Editor Jun Chen and the peer reviewers coming together and discussing their recommendations until a consensus was reached. A fact-based synthesis statement explaining their decision and outlining what is needed to prepare a revision is listed below. The following reviewer(s) agreed to reveal their identity: Edward Hall

Both reviewers acknowledged that this is an interesting study and strong evidence has been provided to support a role of the mTOR/ S6 signaling pathway in mediating adult hippocampal neurogenesis after traumatic brain injury (TBI).

Reviewer #1 indicated that the results are well analyzed, the statistical analysis is appropriate and the data and figures are convincing. Reviewer #2 felt that the manuscript can be further strengthened by including some additional data and a major revision. In specific: 1. (Major) While the pS6 staining is impressive and shows co-localization with Nestin-GFP cells, immunobltting data with the pS6 antibody will add greater credibility of the finding. Since pS6 antibody works well for immunoblotting, the author shall provide this additional piece of data. 2. (Minor) The current figures 1 and 2 should be combined, since they are just different magnification of the same set of data. 3. (Strongly suggested) The current analysis on neurogenesis ends at 48 h post-TBI and BrdU injection, leaving it uncertain why there are truly increased new neuron output-at least one month after BrdU incorporation-following TBI. The paper will be much stronger if the authors can extend their BrdU analysis into BrdU/NeuN labeling comparison at one month after TBI, with or without rapamycin treatment. 4. (Soft suggestion) Will rapamycin treatment attenuate the behavioral outcomes after TBI?

As the reviewing team for your manuscript, we agreed that the suggestions from Reviewer#2 are excellent, and the suggested additional experiments should take less than 2 months to accomplish. We look forward to receiving your revised manuscript that has successfully addressed all points raised in this review.
